# Movement of the stimulated finger in a Go/NoGo task enhances attention directed to that finger as evidenced by P300 amplitude modulation

**DOI:** 10.3389/fnhum.2023.1178509

**Published:** 2023-12-05

**Authors:** Kazuhiro Sugawara, Mayu Akaiwa, Yuya Matsuda, Eriko Shibata, Hidekazu Saito, Takeshi Sasaki

**Affiliations:** ^1^Department of Physical Therapy, School of Health Science, Sapporo Medical University, Sapporo, Hokkaido, Japan; ^2^Graduate School of Health Sciences, Sapporo Medical University, Sapporo, Hokkaido, Japan; ^3^Department of Physical Therapy, Faculty of Human Science, Hokkaido Bunkyo University, Eniwa, Hokkaido, Japan; ^4^Department of Occupational Therapy, School of Health Science, Sapporo Medical University, Sapporo, Hokkaido, Japan

**Keywords:** somatosensory, reaction time, electroencephalography, event-related potentials, P300, movement

## Abstract

Somatosensory cues and the optimal allocation of attentional resources are critical for motor performance, but it is uncertain how movement of a body part modulates directed attention and the processing of somatosensory signals originating from that same body part. The current study measured motor reaction time (RT) and the P300 event-related potential during a required movement response to stimulation of the same body part in a Go/NoGo task under multiple response. In the Movement Condition, participants were instructed to extend their right index finger in response to mild electrical stimulation of the same finger (Go signal) or remain still when receiving electrical stimulation to the fifth right finger (NoGo signal). Movement RTs and P300 amplitudes and latencies were measured under varying Go signal 50% probabilities. In other trial blocks, participants were required to count Go signals but not respond with movement or to ignore all signals while engaged in an unrelated task. Mean RT in the Movement Condition was 234.5 ms. P300 response amplitudes at midline electrodes (Fz, Cz, Pz) were the largest in the Movement Condition. The P300 amplitude at parietal electrode site Pz was significantly greater during Movement Condition trials than during Count Condition trials. The increase in P300 amplitude during trials requiring movement of the same body part receiving somatosensory stimulation suggests that movement itself modulates the attentional resources allocated to that body part.

## 1 Introduction

The execution of fine hand movements requires reliable sensory feedback from both moving and non-moving digits as well as shifts in attentional allocation among these digits, but little is known of how ongoing movement reallocates attentional resources. Reaction time (RT) tasks requiring movement in response to external sensory stimuli (such as visual, auditory, or somatosensory stimuli) have been widely used to evaluate human sensorimotor and attentional functions (Kamitani et al., 2003; Barutchu and Spence, 2021). Several studies have reported that the posterior parietal cortex and premotor cortex participate in the processing of stimulus information for evaluation of response contingencies (Sugawara et al., 2013a,b). Appropriate allocation of attention to stimuli is also critical for efficient response accuracy and/or speed. Event-related potentials (ERPs) triggered by external stimuli and originating from posterior parietal or premotor cortex are thought to reflect the cognitive processing of stimulus–response contingencies (Brazdil et al., 2003). The P300 wave is one of the best-studied ERPs elicited by target stimuli during the oddball task, in which subjects respond to a rare stimulus (target) within a continuous stream of common stimuli. The P300 is characterized by a positive deflection that peaks ∼300–500 ms after stimulus onset and reflects the allocation of attentional resources to that stimulus (Polich and Bondurant, 1997; Kida et al., 2003b; Polich, 2007) regardless of sensory modality (i.e., visual, auditory, or somatosensory) (Duncan-Johnson and Donchin, 1977; Wickens et al., 1983; Johnson, 1986; Nittono et al., 1999; Verleger et al., 2016).

Previous studies measuring motor RTs and ERPs in response to somatosensory stimulation have targeted stimuli mainly to a site contralateral from the movement site (Kida et al., 2003a; Nakata et al., 2004, 2005), while few have examined the effects of movement at the stimulation site (such as the same finger) on attention and somatosensory processing. We conducted a sensory stimulation–motor response task in which somatosensory stimulation was directed to the site of require movement and investigated the change in attention focused on the movement site by measuring the P300. In daily life, fine movements are often cued or guided by somatosensory stimulation. It has been suggested that local somatosensory stimulation and activation of the responsive field in somatosensory cortex (e.g., the finger area) modulates activity of the corresponding primary motor cortex (M1) area and that digit movement reallocates attentional resources (Zentgraf et al., 2009; Rossettini et al., 2017). Kida et al. (2003a) reported that P300 was modulated by external stimulation and movement during a Button-pressing condition compared to a non-movement (Count) condition. However, few studies have examined how movement site and the differences as due to the presence or absence of a motor plan for the same finger influences somatosensory processing (Kida et al., 2003b; Nakata et al., 2004).

In this study, we assessed the change in P300 during somatosensory stimulation of a finger required to move in response to that stimulus. Our hypothesis is that attention will be reallocated to a stimulated finger that is also the required movement finger as evidenced by increased P300 amplitude in the Movement Condition compared to a no-Movement Condition (Count and Ignore conditions).

## 2 Materials and methods

### 2.1 Participants

The participants in this study were 15 healthy young adults {age [mean ± standard deviation (SD)], 22.7 ± 1.7 years; all right-handed; 10 males, 5 females}, all of whom provided written informed consent. None of the participants engaged in recreational drug use or used psychotropic medication that affected the central nervous system. The study was conducted in accordance with the tenets of the Declaration of Helsinki and the Code of Ethics of the World Medical Association and was approved by the ethics committee of Sapporo Medical University (No. 30-2-44).

### 2.2 Experimental procedures

The Go/NoGo task included three trial conditions: Movement, Count, and Ignore. During the task, participants rested their arms comfortably on the armrest of a plastic table with the elbow joint flexed 45–50 degrees, hands in full pronation, and digits extended naturally. Subjects were instructed to keep their eyes open and to look at a fixation point approximately 1 m away. In the Movement Condition, the participants performed a simple sensorimotor response task in which they extended their right index finger as quickly as possible after (Go) stimulation of that same finger. Alternatively, all fingers were to remain still in response to stimulation of the right fifth finger. In the Count Condition, the participants silently counted Go stimuli with no-movement, and were given feedback on count accuracy at the end of each block (200 trials) to maintain attention and accuracy. In the Ignore Condition, the participants were asked to silently read numbers presented on a PC display and to ignore all other stimuli. The Go and NoGo stimulus probabilities, 50: 50, were set for each response condition. Participants completed one block for each response condition (3 blocks in total). The inter-stimulus interval was set at 2 s for all conditions. At least 60 EEG responses were averaged for each response condition, and blocks were presented in pseudorandom order on the same day. All participants performed a 2-min, 60-trial practice block prior to recordings, 30 required motor responses.

### 2.3 Stimulation

The participants wore ring electrodes on the index and fifth fingers of the right hand for electrical stimulation. At both sites, the electrical stimulus was a 0.2-ms constant current square-wave pulse at three times the sensory threshold, which yielded no pain or unpleasant sensations. The anode was placed at the distal interphalangeal joint and the cathode at the proximal interphalangeal joint. The index finger was stimulated for the Go condition and the fifth finger for the NoGo condition.

### 2.4 Electroencephalography (EEG) recordings

All electrophysiological recordings were acquired using the Neuropack system (Nihon Kohden, Tokyo, Japan). Electroencephalograms were recorded with Ag/AgCl disk electrodes placed on the scalp at Fz, Cz, and Pz according to the International 10–20 System. Each scalp electrode was referenced to the linked earlobes (A1A2) and impedance was maintained at less than 5 kΩ. Signals were recorded to computer at a sampling rate of 1,000 Hz and band-pass filtered at 0.1–200 Hz. An electrooculogram (EOG) was recorded from the right suborbital region to monitor eye movements or blinks exceeding 100 μ (Kida et al., 2003b). Electroencephalographic recordings with accompanying EOG signals > 100 μV and those contaminated by noise or other non-ocular artifacts greater than ± 200 μV were removed from the analysis. For each recording, a 100-ms baseline and 600-ms post-stimulus epoch were used for ERP analysis. P300 peak amplitudes (baseline-to-peak measurement), which were determined from individual ERPs, were measured within time windows 250–650 ms (Kida et al., 2004; Verleger et al., 2016). Slow responses exceeding 500 ms in duration and responses on incorrect trials (Go on NoGo or vice versa) were eliminated before averaging and analysis.

### 2.5 Electromyography (EMG)

The EMG was measured using a pair of Ag/AgCl electrodes (Blue-sensor NF-00; Ambu, Denmark) mounted over the right extensor indicis muscle at a distance of 2 cm. The EMG signals were sampled at 1,000 Hz (Power Lab; AD Instruments) and band-pass filtered at 0.1–200 Hz. We calculated RT as the time from right index finger (Go) stimulus onset to right extensor indicis muscle EMG onset (defined as the point at which the rectified EMG exceeded two standard deviations above baseline) (Sugawara et al., 2013b).

### 2.6 Statistical analysis

All data are expressed as the mean ± SD. The Shapiro–Wilk test was used to assess dataset normality, with *P* < 0.001 accepted as significant, and Mauchly’s test to assess the assumption of sphericity. If the sphericity assumption was violated, the Greenhouse–Geisser adjustment was used for correction. For the peak amplitude and latency of P300, two-factor analysis of variance with repeated measures was performed [response condition (Movement, Count, Ignore) ⋅ electrode (Fz, Cz, Pz)] within-subject factors. *Post-hoc* tests were performed in cases with significant differences, with Bonferroni correction for multiple comparisons. Significance was set at *P* < 0.05 (corrected), and all statistical calculations were performed using SPSS version 24 (IBM, Armonk, NY).

## 3 Results

More than 100 trials were obtained for each condition. The average number of trials included were as follows: Movement/Go 281.6 ± 16.4 times; Count/Go 296.6 ± 9.6 times; Ignore/Go 294.2 ± 7.3 times. Mean RTs in the Movement Condition were 234.5 ± 46.3 ms. Error rates was 2.4% in the Movement Condition and was 0.9% in the Count Condition.

**FIGURE 1:**
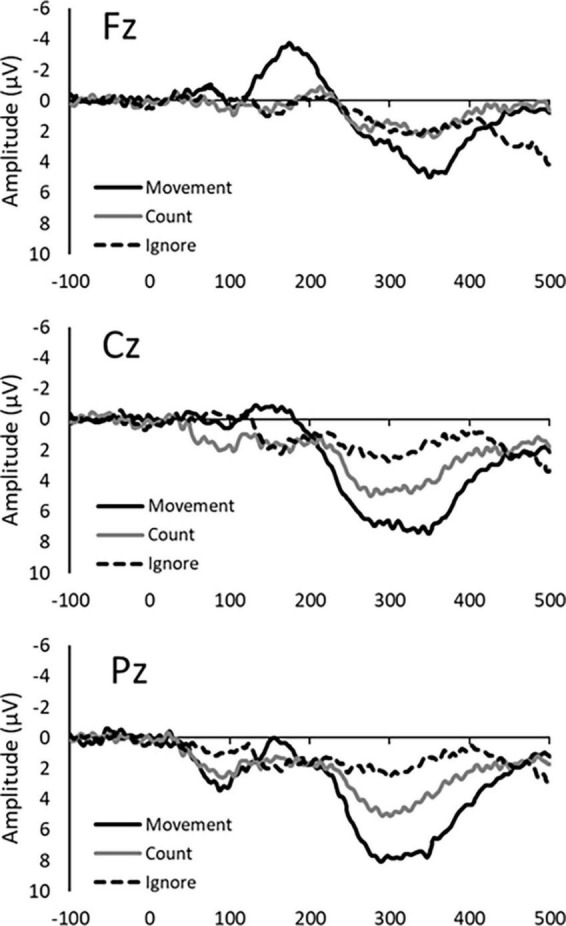
Average ERP waveforms in a representative participant for each response condition (Movement, Count, and Ignore) at the Fz, Cz, and Pz electrode site.

The P300 was evoked by three response conditions. Average amplitudes and latencies at Fz, Cz, and Pz electrode sites are summarized in Tables 1, 2, while Figure 1 depict the average ERP waveforms in a representative participant at Fz, Cz, and Pz, respectively, for each response condition. Two-way repeated-measures ANOVA revealed a significant response condition × electrodes interaction effect on P300 amplitude [F(4, 56) = 8.224, *p* < 0.001], In addition, The main effect of the response condition was also significant [F(2, 28) = 17.342, *p* < 0.001]. *Post hoc* analysis revealed that P300 amplitude was significantly larger in the Movement and Count Conditions than the Ignore Condition at Cz and Pz (*p* < 0.05). The P300 amplitude was also significantly larger in the Movement Condition than the Count Condition at Pz (*p* = 0.018) (Table 2). Two-way repeated-measures ANOVA revealed no significant response condition × electrodes interaction effect on P300 latency [F(4, 56) = 0.470, *p* = 0.758]. In addition, the main effect of the response condition was no significant [F(2, 28) = 2.678, *p* = 0.086]. The main effect of the electrode was also no significant [F(2, 28) = 2.007, *p* = 0.153]. However, for all electrodes, the latency of the Movement Condition tended to be delayed compared to the latency of the other Conditions (Table 2).

**TABLE 1:** The average amplitude (μV) for P300 in each Condition.

Amplitude (μV)	Movement	No-Movement	
		Count	Ignore
Fz	6.1 (4.9)	5.0 (2.0)	2.4 (1.3)
Cz	9.3 (6.2)	8.7 (2.7)	3.5 (1.6)
Pz	10.4 (4.3)	7.4 (2.4)	2.7 (1.2)

Data are expressed as means (standard deviations).

**TABLE 2:** The average latency (ms) for P300 in each Condition.

Latency (ms)	Movement	No-Movement	
		Count	Ignore
Fz	322.7 (45.7)	314.7 (35.1)	298.6 (33.7)
Cz	318.4 (37.0)	300.7 (22.4)	295.7 (30.5)
Pz	314.5 (31.4)	308.9 (27.3)	294.7 (40.8)

Data are expressed as means (standard deviations).

## 4 Discussion

We used a somatosensory Go/NoGo task to investigate the effects of response demands (movement, counting, or ignoring) on P300 potentials as indices of processing attentional allocation. In the Movement Condition, at midline electrodes Fz, Cz, and Pz, P300 amplitude increased. At the parietal site Pz, P300 amplitude and delay were modulated by response type, with higher amplitudes and longer latencies in Movement blocks (when subjects were required to move the stimulated finger) compared to the Count Condition (in which subjects were required to silently count Go stimuli). These findings indicate that movement enhances the attention allocated to the movement site.

The P300 amplitude was also greater in the Movement Condition and Count Condition than the Ignore Condition. According to several previous studies, P300 amplitude is positively associated with attentional allocation (Wickens et al., 1983; Yagi et al., 1999; Polich, 2007). However, this association has not been consistent across studies, with some reporting larger P300 in the Hand-movement Condition compared to the Count Condition (Johnson, 1986; Kotchoubey, 2014), but others reporting larger P300 in the Count Condition than the Hand-movement Condition (Barrett et al., 1987) or no difference between conditions (Starr et al., 1995). However, these studies did not address the change in P300 when the same body part required to move in response to somatosensory stimulation was also the target of Go stimulation. It has been reported that focused attention on external stimuli improves movement performance (Zentgraf et al., 2009). In the present study, it is possible that both attention to sensory stimulation to the index finger and expected movement, extension of the index finger, were required internally focused attention. Somatosensory input was also found to enhance the performance of complex finger movements (Rothwell et al., 1982; Blennerhassett et al., 2007). Therefore, when a stimulated body part is also the moving body part, attention to somatosensory stimuli may be modulated not only by the somatosensory cortex extending to the motor area but also by movement (i.e., movement influences attention).

Also consistent with previous studies (Polich and Bondurant, 1997; Nakajima and Imamura, 2000; Kida et al., 2003b), P300 latency was prolonged at lower Go stimulus probabilities. Peak latency is regarded as an important index of stimulus classification speed or stimulus evaluation time in choice RT tasks (Kutas et al., 1977; Magliero et al., 1984; Thorpe et al., 1996). Kida et al. (2003b) reported that P300 latency was delayed in an oddball task using median nerve stimulation among participants who exhibited longer RTs (Kida et al., 2003b). We found longer latency of P300 concomitant with greater P300 amplitude in Movement Condition, suggesting that stimulus evaluation time and attentional allocation increase for movement in response to stimuli.

This study has several limitations. We did not include a comparison condition where the “Go” signal was delivered to a different finger as this study focused on the relationship between movement and attention. Therefore, P300 was compared only between movement and no-movement conditions in response to the same Go stimulus. Since we found that attention was increased by movement, we need to clarify whether the same results are observed when the “Go” signal is delivered to a different (non-movement) finger or since the difficulty of moving each finger is different, it may be better to perform the button-pressing task with the mother finger or with the extremities, as used in previous studies. We also did not measure movement-related cortical potentials (MRCPs) during the Movement Condition, and it is possible that P300 waveforms include this activity because the movement RTs were generally within the P300 time window. MRCPs is composed of the Bereitschaftspotential (readiness or premotor potential) which is observed before movement onset, and post-movement component after movement onset (Shibasaki et al., 1980; Tarkka and Hallett, 1991; Nagamine et al., 1996). The Bereitschaftspotential is generally observed as a negative potential, and P300 was observed as a positive potential. Therefore, if a (negative) movement-related signal is superimposed onto P300, it would be expected to reduce P300 amplitude, while P300 amplitude was enhanced in the Movement Condition at all Go stimulus probabilities. However, since post-movement component of MRCP is observed after approximately 350 ms (Neshige et al., 1988; Kristeva et al., 1990), the post-movement component of MRCP may overlap with P300 in participants with slower RT.

## 5 Conclusion

These results suggest that movement in response to somatosensory stimulation of the same body part shifts the allocation of attention to that body part.

## Data availability statement

The datasets presented in this study can be found in online repositories. The names of the repository/repositories and accession number(s) can be found in this article/supplementary material.

## Ethics statement

The study was conducted in accordance with the tenets of the Declaration of Helsinki and the Code of Ethics of the World Medical Association and was approved by the Ethics Committee of Sapporo Medical University (No. 30-2-44). The studies were conducted in accordance with the local legislation and institutional requirements. The participants provided their written informed consent to participate in this study.

## Author contributions

Material preparation and data collection were performed by KS, MA, YM, ES, and TS. Formal analyses were performed by YM, HS, and KS. The first draft of the manuscript and the review and editing of the manuscript were written by KS. All authors contributed to the study’s conception and design, commented on previous versions of the manuscript, read, and approved the final manuscript.
